# Myocarditis: A Rare Complication of Immune Checkpoint Inhibitor Therapy

**DOI:** 10.7759/cureus.60459

**Published:** 2024-05-16

**Authors:** Devi Parvathy Jyothi Ramachandran Nair, Shilla Zachariah, David Scollan, Atif Shaikh

**Affiliations:** 1 Internal Medicine, Tower Health Medical Group, Reading, USA; 2 Cardiology, Tower Health Medical Group, Reading, USA

**Keywords:** heart failure, non-ischemic cardiomyopathy, myocarditis, pembrolizumab, immune checkpoint inhibitor

## Abstract

Immune checkpoint inhibitors (ICIs) are a class of immunotherapy agents that are often used in cancer treatment. A rare but life-threatening complication that can be seen is ICI-induced myocarditis. We discuss a case of pembrolizumab-induced myocarditis and the nuances involved in timely diagnosis and treatment. A 64-year-old female with a past medical history significant for metastatic right-sided colorectal adenocarcinoma undergoing immunotherapy with pembrolizumab presented with worsening shortness of breath and was found to be hypoxic. Initial laboratory analysis was remarkable for troponin of 0.35 ng/mL, which later peaked at 6.01 ng/mL. The electrocardiogram showed non-specific ST segment changes in the anteroseptal leads, and a subsequent echocardiogram revealed severely reduced left ventricular systolic function with an ejection fraction of 25%. Coronary angiography showed non-obstructive coronary arteries. As the patient was on pembrolizumab immunotherapy for cancer, there was high suspicion of ICI-induced myocarditis, and the patient was started empirically on steroids. Subsequently, cardiac magnetic resonance imaging was done, which confirmed the diagnosis of myocarditis. Pembrolizumab therapy was discontinued, and she was started on guideline-directed medical therapy for heart failure. While ICIs have transformed cancer therapy, healthcare providers must be vigilant for immune-related adverse events such as myocarditis. Early recognition, prompt management, and close monitoring are crucial for optimizing patient outcomes.

## Introduction

Immune checkpoint inhibitors (ICIs) are a class of immunotherapy agents that have revolutionized cancer treatment by enhancing the immune system's ability to recognize and attack cancer cells. They are a class of monoclonal antibodies that target host immune negative regulation receptors, such as CTLA‐4 (cytotoxic T‐lymphocyte-associated protein 4), programmed cell death receptor 1 (PD‐1), and programmed cell death ligand 1 (PD‐L1) [1]. Some of the US Food and Drug Administration-approved ICIs include ipilimumab (anti-CTLA-4), nivolumab, pembrolizumab, cemiplimab (anti-PD-1), avelumab, atezolizumab, and durvalumab (anti-PD-L1). With ICI therapy becoming more prevalent, we have started to notice many immune-related adverse events. ICIs break the balance of the body's immune system and reduce T-cell tolerance, thereby leading to the production of a series of adverse events. [2]. These affect various organ systems in the body, including the skin, digestive, endocrine, respiratory, hematological, renal, and rarely the nervous and cardiovascular systems. Cardiac toxicities are rare and can range from pericarditis causing pericardial effusion to myocarditis and even severe fatal heart failure [3]. Symptoms can present from shortly after initiation to several months after discontinuation. We report the case of pembrolizumab-induced myocarditis presenting as heart failure in a patient with colonic adenocarcinoma.

## Case presentation

A 64-year-old female with a past medical history significant for metastatic right-sided colorectal adenocarcinoma, embolic stroke in the setting of malignancy, chronic obstructive pulmonary disease, hypertension, type 2 diabetes mellitus, and hyperlipidemia presented to the emergency department with worsening of shortness of breath over one week. She reported that she initially started noticing shortness of breath with exertion; however, for the past 3 or 4 days, she has been getting short of breath even at rest, which prompted her to come to the hospital. She denied any fever, chest pain, palpitations, lightheadedness, or symptoms of recent viral illnesses. On the physical exam, heart sounds were tachycardic and regular. Lungs were noted to have wheezes to auscultation bilaterally. No jugular venous distention or lower extremity edema was noted. Pulse oximetry initially showed an oxygen saturation of 70%, requiring 10 liters of supplemental oxygen via nasal cannula to maintain saturation above 90%.

Laboratory analysis (Table 1) was remarkable for elevated troponin of 0.35 ng/mL, creatine kinase (CK) of 687 IU/L, and a white blood cell count of 13.8 x 103/µL. The respiratory viral panel was negative. ﻿

**Table 1:** Laboratory analysis Table shows relevant laboratory test results and reference ranges.

Laboratory test	Value	Reference Range
Sodium (mmol/L)	141	136-145
Potassium (mmol/L)	4.5	3.5-5.1
Chloride (mmol/L)	104	98-107
Blood Urea Nitrogen (mg/dL)	19	7-25
Creatinine (mg/dL)	1.11	0.6-1.3
Glucose (mg/dL)	252	70-99
Calcium (mg/dL)	9.6	8.6-10.3
Magnesium (mg/dL)	2	1.9-2.7
Albumin (g/dL)	4.3	3.5-5.7
White blood cell (x10^3^/µL)	13.8	4.8-10.8
Red blood cell (x10^6^/µL)	4.73	4.5-6.1
Hemoglobin (g/dL)	14.4	12-16
Platelets (x10^3^/µL)	251	130-400
Troponin (ng/mL)	0.35	<=0.06
Brain natriuretic peptide (pg/mL)	25	0-100
Creatine kinase (IU/L)	687	30-223

An electrocardiogram (ECG) revealed non-specific ST segment elevations in anteroseptal leads with no reciprocal changes. A computed tomography angiography of the chest was negative for pulmonary embolism.

The patient received aspirin 325 mg with a subsequent dosing of 81 mg daily and was started on an IV heparin infusion. Troponin levels peaked at 6.01 ng/mL. The echocardiogram revealed reduced left ventricular systolic function with an ejection fraction (EF) of 25%, hyperdynamic basal segments, and severe hypokinesis of the remaining segments. An echocardiogram that was done about six months ago showed normal left ventricular function with an EF of 56%, normal diastolic function, and no regional wall motion abnormalities. A left-heart catheterization performed revealed nonobstructive coronary artery disease.

**Figure 1:**
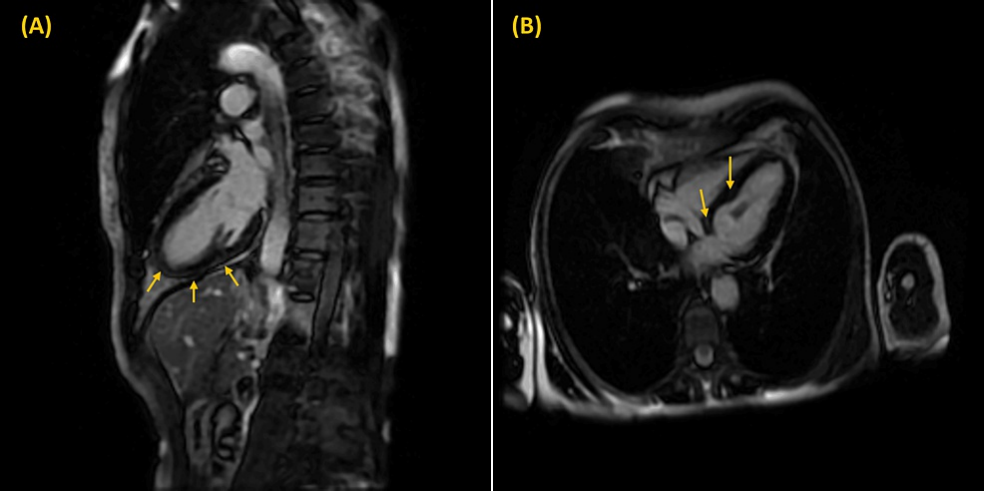
Cardiac magnetic resonance imaging Arrows in (A) indicate subtle delayed enhancement at the subepicardial region at the inferior wall extending from base to apex; arrows in (B) indicate subtle delayed enhancement at the subepicardial to midmyocardium region at the septum at the base, mid-level, and apex.

On further reviewing the history, the patient had completed five cycles of pembrolizumab (200mg intravenous (IV) infusion every six weeks), with the last dose approximately four weeks prior. Given the potential concern for ICI-induced myocarditis, the patient was empirically started on IV methylprednisolone 1 gram daily. A subsequent cardiac magnetic resonance imaging (CMR) scan revealed subepicardial late gadolinium enhancement at the inferior wall and septum, suggesting the diagnosis of myocarditis (Figure 1). 

She was continued on IV methylprednisolone (1 gram daily) for three days, followed by a long oral prednisone taper. Pembrolizumab therapy was discontinued after discussion with her oncologist. She was started on guideline-directed medical therapy for heart failure with metoprolol succinate, lisinopril, empagliflozin, and spironolactone, and was discharged home with a wearable defibrillator. She was scheduled for outpatient follow-up in the heart failure clinic in one week and with her outpatient oncologist in one week. A repeat echocardiogram after three months of guideline-directed medical therapy showed recovered left ventricular systolic function with an EF of 65% and resolution of wall motion abnormalities.

## Discussion

This patient presented with symptoms of heart failure and was diagnosed with myocarditis. Immune checkpoint inhibitor-induced myocarditis is a relatively rare but severe and life-threatening complication. ICI-related myocarditis has a reported incidence of 0.04% to 1.14% [1]. Symptoms can present from shortly after initiation to several months after discontinuation of the ICI therapy. We discuss the nuances involved in the timely diagnosis and treatment of a rare case of ICI-induced myocarditis.

Cardiotoxicity secondary to ICIs can present as myocarditis, pericarditis, heart failure, arrhythmias, and vasculitis, even without underlying cardiac risk factors [4]. Myocarditis has the highest mortality rate among all types of ICI-induced cardiotoxicity, with a mortality rate approaching 26.2% [5]. Patients can present with symptoms such as chest pain, shortness of breath, fatigue, palpitations, and, in severe cases, heart failure.

Diagnosis can be made with diagnostic testing, including ECG, troponin, brain natriuretic peptide (BNP), creatine kinase (CK), and cardiac imaging with echocardiography, coronary angiography, and cardiac magnetic resonance imaging (CMR). BNP, troponin, and CK levels are usually elevated. ECG findings can range from premature atrial or ventricular contractions to ventricular tachycardia or complete heart block [6]. Echocardiogram findings can range from normal function to decreased systolic function with or without regional wall motion abnormalities and the presence of a pericardial effusion [6,7]. CMR is noted to be superior to echocardiography in the diagnosis of myocarditis [6]. When CMR is not available or safe, cardiac fluorodeoxyglucose positron emission tomography can be used to assess inflammation. The gold standard for diagnosis remains endomyocardial biopsy (EMB); however, EMB is usually undertaken in cases where the non-invasive tests are inconclusive, but strong clinical suspicion remains. Current diagnostic criteria have been classified into three groups: definite myocarditis, probably myocarditis, and possible myocarditis based on clinical symptoms, diagnostic lab work, and imaging [1] (Table 2).

**Table 2:** Classification criteria for myocarditis [1] CMR: cardiac magnetic resonance imaging, CAD: coronary artery disease, ECG: electrocardiogram, WMA: wall motion abnormality. The information presented in this table is obtained from an article published under a Creative Commons License.

Definite myocarditis: ≥1 of the following	Probably myocarditis	Possible myocarditis
Pathology consistent with myocarditis	Diagnostic CMR without clinical syndrome of myocarditis, positive ECG, or positive biomarker, OR	Suggestive CMR without clinical syndrome of myocarditis, positive ECG, or positive biomarker, OR
Diagnostic CMR, clinical syndrome of myocarditis, and positive biomarker or ECG	Suggestive CMR with one of the following: Clinical syndrome of myocarditis Positive ECG Positive biomarker, OR	Echocardiography with WMA and clinical syndrome of myocarditis or positive ECG, OR
Echocardiography with WMA, clinical syndrome of myocarditis, positive biomarker, positive ECG, and negative angiography for CAD	Echocardiography with WMA and clinical syndrome of myocarditis with either positive ECG or biomarker, OR	Elevated biomarker with clinical syndrome of myocarditis or positive ECG and no alternative diagnosis.
-	Clinical syndrome of myocarditis with positron emission tomography scan evidence and no alternative diagnosis	-

The management of ICI-induced myocarditis mainly relies on insights from past case reports and case series due to a lack of randomized controlled trials or prospective studies [8]. Per American Society of Clinical Oncology (ASCO) guidelines, ICI-induced myocarditis can be divided into four grades: Grade 1 (abnormal cardiac biomarkers with no symptoms and no ECG abnormalities), Grade 2 (abnormal cardiac biomarkers with mild symptoms or new ECG abnormalities without conduction delay), Grade 3 (abnormal cardiac biomarkers with either moderate symptoms or new conduction delay), and Grade 4 (moderate to severe decompensation) [9]. ICI should be immediately held for Grade 1 myocarditis and permanently discontinued for Grade 2-4 myocarditis [8]. High-dose corticosteroids are typically used as the first-line treatment to suppress the immune response and reduce inflammation. 1-2 mg/kg of prednisone intravenously or orally can be given, and if the symptoms are refractory, IV methylprednisolone 500-1000 mg daily can be considered [10]. If there is no response to steroids, immunosuppressants such as mycophenolate mofetil or infliximab or anti-transplant rejection medications such as anti-thymocyte globulin can be considered [10,11].

## Conclusions

There are a few key takeaways from this case report, one of which is the importance of being aware of the immune-related adverse events associated with pembrolizumab and other immune checkpoint inhibitor therapies and having a high suspicion for a rare but life-threatening complications like myocarditis in patients presenting with cardiac symptoms who are undergoing ICI therapy. Also, it is important to closely monitor the cardiac function and clinical status during and after treatment with ICIs, as complications can occur soon after treatment or months after its discontinuation. Finally, this case report highlights that early recognition of symptoms, diagnosis, and treatment are critical to preventing morbidity and mortality associated with adverse events of ICI therapy.
